# Compensatory roles of CD8^+^ T cells and plasmacytoid dendritic cells in gut immune regulation for reduced function of CD4^+^ Tregs

**DOI:** 10.18632/oncotarget.7510

**Published:** 2016-02-19

**Authors:** Young-In Kim, Bo-Ra Lee, Jae-Hee Cheon, Bo-Eun Kwon, Mi-Na Kweon, Hyun-Jeong Ko, Sun-Young Chang

**Affiliations:** ^1^ College of Pharmacy, Ajou University, Suwon, Korea; ^2^ College of Pharmacy, Kangwon National University, Chuncheon, Korea; ^3^ Department of Internal Medicine and Institute of Gastroenterology, Yonsei University College of Medicine, Seoul, Korea; ^4^ Mucosal Immunology Laboratory, Department of Convergence Medicine, Asan Medical Center, University of Ulsan College of Medicine, Seoul, Korea

**Keywords:** gut, CD4^+^ Tregs, CD8^+^ T cells, plasmacytoid dendritic cells, IL-10, Immunology and Microbiology Section, Immune response, Immunity

## Abstract

CD4^+^ Tregs need to migrate from the mucosal periphery into the draining lymph node via CCR7 to exert their suppressive effects. In this study, we investigated whether CCR7 deficiency resulted in failure of immune suppression in 2% dextran sulfate sodium-induced colitis. Unexpectedly, intestinal inflammation was not exacerbated in the absence of CCR7. Expression of IL-10, a representative suppressive cytokine, was enhanced in CCR7KO CD8^+^ T cells. Colon CCR7KO CD8^+^ T cells reduced the activation of CD4^+^ T cells. Depletion of CD8^+^ T cells using anti-CD8 antibody exacerbated colitis in CCR7KO mice. Plasmacytoid dendritic cell numbers were also slightly increased during intestinal inflammation in the absence of CCR7, and the depletion of those cells exacerbated DSS-induced colitis in CCR7KO mice. These results suggest that CD8^+^ T cells and plasmacytoid dendritic cells have compensatory roles in immune regulation in the gut for impaired function of CD4^+^ Tregs.

## INTRODUCTION

In mammals, the highest microbial load in the body is in the digestive tract, which harbors up to 10^14^ commensal bacteria per gram of fecal matter [1]. The intestinal immune system keeps commensal microbes at bay by the secretion of mucin, antibacterial substances, and secretory IgA as well as by tightening of the epithelial barrier [2, 3]. Commensal microbiota competes with and inhibits pathogens, thus contributing to the host's defense against infection [4]. Thus, it seems that the gastro-intestinal immune system sustains the intestinal microbiota to maintain a mutually beneficial state of intestinal homeostasis [1]. However, a breakdown of immune regulation can result in aberrant inflammatory responses such as in the case of inflammatory bowel diseases (IBD) [5].

IBD is a group of inflammatory conditions affecting the small and large intestine in man, including ulcerative colitis (UC) and Crohn's disease (CD) [6]. This is a complex disease caused by the interaction of environmental and genetic risk factors. IL-17-producing Th17 cells play a key pathogenic role in chronic inflammatory conditions [7]. To suppress inflammation, several regulatory T cells (Tregs) inhibit the differentiation and function of pathogenic effector T cells. Foxp3^+^CD4^+^ Tregs make a significant contribution to immune suppression in the gut immune system [8]. Foxp3 expression is essential for differentiation and suppressor function of CD4^+^ Treg cells and controls the development of both, naturally occurring and antigen-induced Treg cell lineage [9]. Foxp3^−^CD4^+^ Tregs, such as Tr1 and Th3, also play a suppressive role in mucosal tissue [10]. Depletion of Foxp3^+^ Treg exacerbates intestinal inflammation even in the T-cell-independent colitis model [11].

CC chemokine receptor 7 (CCR7) is highly expressed on several immune cells such as naïve and central memory T cells, B cells, and mature dendritic cells (DCs) [12]. CCR7 ligands, CCL21 and CCL19, are constitutively produced in the lymph node and their interaction with CCR7 induces the migration of CCR7-bearing immune cells to the lymph nodes [13]. CCR7 deficiency results in the attenuation of immune responses due to the impaired migration of DCs and abnormal secondary lymphoid organ architecture [12]. CCR7 is also required for the return of T cells from the peripheral tissue to the lymph nodes [14]. The Foxp3^+^CD4^+^ Treg cells, also expressing CCR7 [15], migrate from the periphery towards the lymph nodes to suppress the induction of immune responses [16]. Thus, lack of CCR7 expression leads to defective lymph node localization and consequent functional impediment of Foxp3^+^CD4^+^ Tregs. As a result, CCR7KO Tregs are less effective in regulation of colitis [16]. Thus, CCR7 critically controls the *in vivo* function of CD4^+^ Tregs by mediating their localization in the appropriate tissue.

Here, we investigated if CCR7 deficiency aggravates DSS-induced colitis. We hypothesized that CCR7 deficiency resulted in functional defect of CD4^+^ Tregs, leading to severe intestinal pathogenesis in response to inflammatory stimuli. Unexpectedly, CCR7KO mice had less severe inflammation in the gut when compared with wild-type (WT) mice, although CCR7KO CD4^+^ Tregs showed impaired migration to the lymph nodes. To explain the resistance to DSS-induced colitis in CCR7KO mice, we analyzed various immune cells and the expression of different cytokines to determine other factors that suppress immune responses in the gut.

## RESULTS

### CCR7 deficiency did not exacerbate DSS-induced colitis

**Figure 1:**
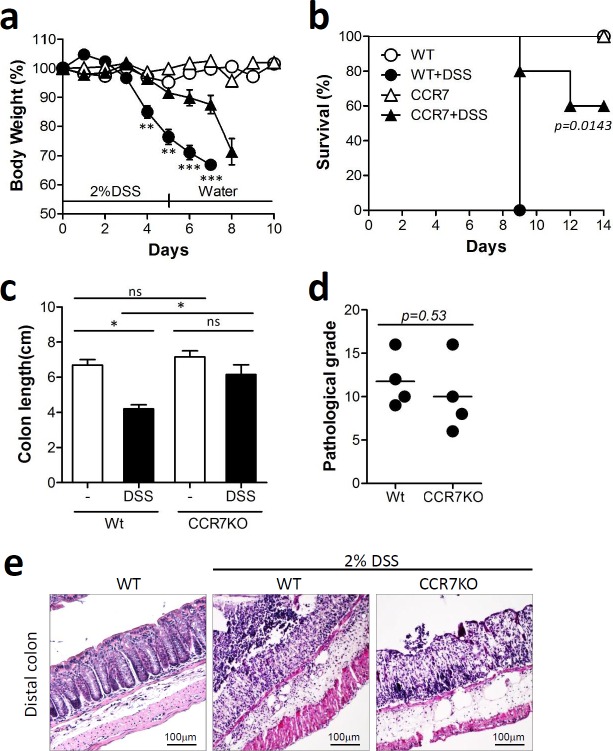
CCR7 deficiency did not exacerbate DSS-induced colitis Mice were treated with 2% DSS for 5 days followed by switching to drinking water. Data are representative of three independent experiments. **a.** Body weight changes. Student's *t*-test. **p < 0.01, ***p < 0.001 compared between WT DSS *vs* CCR7KO DSS groups. **b.** survival rate. Log-rank (Mantel-Cox) test, compared between WT DSS *vs* CCR7KO DSS groups **c.** colon length; ns, not significant; *p < 0.05; one-way ANOVA, **d.** pathological grade from histological examination of colon stained with H&E; Student's *t*-test, (e) representative H&E images (*n* = 5).

Foxp3^+^CD4^+^ Tregs are known to play a major role in immune suppression in the intestine [8]. In the absence of CCR7, Tregs as well as naïve T cells are unable to migrate from the mucosal periphery into the draining lymph nodes and therefore fail to exert their regulatory effect. In this regard, CCR7-deficient Treg cells are less capable of inhibiting intestinal inflammation *in vivo* [16]. CCR7 deficient mice develop diarrhea autoimmune gastritis and exocrinopathy accompanied by the formation of mucosal tertiary lymphoid follicle which causes diarrhea associated with altered ion transport in colonocytes in absence of overt colitis [17]. Here, we investigated whether CCR7 deficiency leads to severe intestinal inflammation in a murine dextran sulfate sodium (DSS)-induced colitis model. Wild-type C57BL/6 (WT) and CCR7-knock out (CCR7KO) mice were treated with 2% DSS in drinking water for 5 days and then switched to normal drinking water thereafter. Body weight and survival rate of mice was monitored in both groups. Unexpectedly, CCR7KO mice showed slightly alleviated weight loss (Figure 1a) and longer survival time after severe inflammatory disease, compared with WT mice (Figure 1b). Although there was no significant difference of colon length in WT and CCR7KO mice at steady state, the colon length of WT mice was significantly decreased than that of CCR7KO mice after severe inflammatory disease (Figure 1c). Further, histological examination of colon showed that there was no significant difference in the pathological grade between WT and CCR7KO mice after DSS treatment (Figure 1d and 1e). Collectively, these data suggested that DSS-induced colitis was not aggravated in CCR7KO mice in comparison with WT mice, despite the immobilization of Foxp3^+^ Tregs.

### Infiltration of innate immune cells in DSS-induced colitis slightly reduced in absence of CCR7

**Figure 2:**
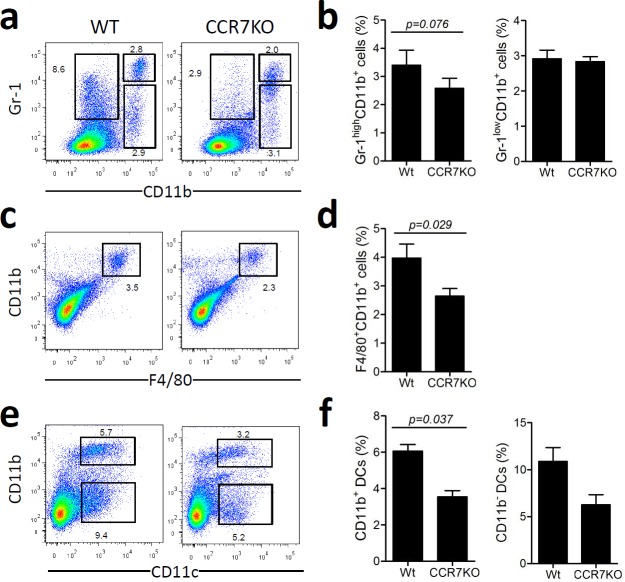
Infiltration of innate immune cells in DSS-induced colitis slightly reduced in the absence of CCR7 At Day 8 of DSS-induced colitis, innate immune cells were analyzed from the colon (*n* = 3). Data are representative of three independent experiments. **a.-b.** Gr-1^+^CD11b^+^ neutrophil population analysis, **c.-d.** F4/80^+^CD11b^+^ macrophages analysis, **e.-f.** dendritic cell analysis. Student's *t*-test.

To assess inflammation in the colon, infiltrated immune cells were analyzed at Day 8 of DSS-induced colitis. There was a slight but insignificant decrease in the number of CD11b^+^Gr-1 ^high^ neutrophils in CCR7 KO mice (Figure 2a and 2b). The numbers of CD11b^+^F4/80^+^ macrophages and CD11c^+^CD11b^+^ dendritic cells (DCs) were lower in CCR7 KO mice than in the WT mice (Figure 2c-2f). However, populations of Gr-1^low^CD11b^+^ myeloid cells and CD11c^+^CD11b^−^ DCs were not significantly different in the two groups. These data suggested that the colon in CCR7KO mice had less infiltration of innate immune cells, a representative marker of inflammation, than that in the WT mice with DSS-induced colitis.

### CD4^+^ Tregs accumulated in the gut in the absence of CCR7

**Figure 3:**
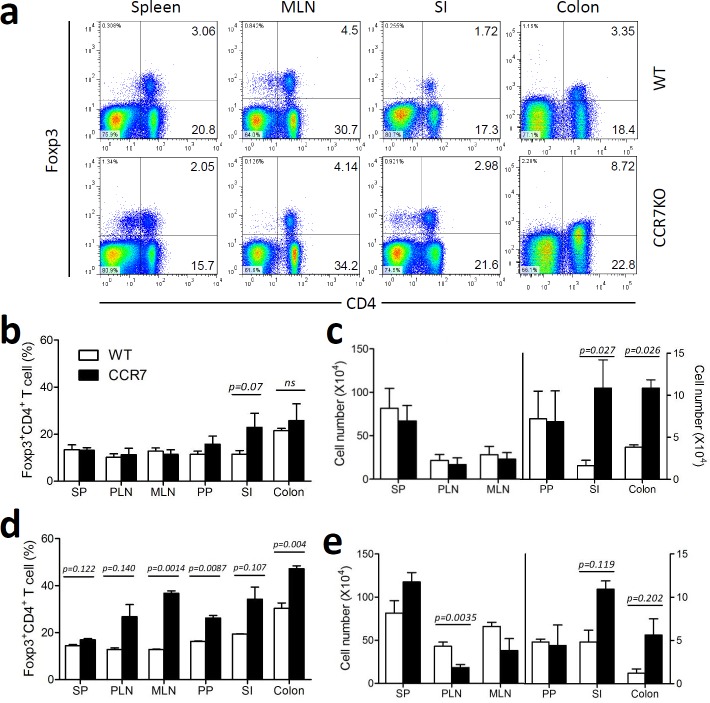
CD4^+^ Treg accumulation in the gut in the absence of CCR7 Foxp3^+^CD4^+^Treg were analyzed in the lymphoid tissue and peripheral gut tissues. **a.** representative plots, **b.** the percentages and **c.** absolute cell number of Foxp3^+^CD4^+^ Tregs from WT or CCR7KO mice under steady state conditions, **d.** the percentage and **e.** absolute cell number of Foxp3^+^CD4^+^ Tregs from WT or CCR7KO mice at Day 8 of DSS-induced colitis (*n* = 4). Student's *t*-test.

Based on the attenuated colitis shown in DSS-treated CCR7KO mice, we verified whether migration of CD4^+^ Tregs into the lymph node was impaired in the absence of CCR7. To assess this, we analyzed Foxp3^+^CD4^+^ Tregs from various sites of the lymphoid and peripheral gut in CCR7KO mice in comparison to that of WT mice. At the steady state, there were no significant differences in the percentage and absolute number of Foxp3^+^CD4^+^ Tregs from the SP, PLN, MLN, and PP between WT and CCR7KO mice (Figure 3a-3c). However, in the intestine, especially the SI, CCR7KO mice showed an increase in ratio and number of Foxp3^+^CD4^+^ Tregs, suggesting the possibility of peripheral accumulation of Tregs (Figure 3a-3c). During DSS-induced colitis, CCR7KO mice showed a higher percentage of Foxp3^+^CD4^+^ Tregs in every tissue sampled than the WT mice. However, the numbers of Foxp3^+^CD4^+^ Tregs in the PLN and MLN of CCR7KO mice were lower than those in the WT mice, which was probably due to the impaired migration of CCR7KO Tregs or dendritic cells from peripheral tissues into the lymph nodes (Figure 3d and 3e). In contrast, CD4^+^ Tregs were accumulated in the intestines of CCR7KO mice in the SI as well as in the colon. Foxp3^+^CD4^+^ Tregs were analyzed further, based on their surface expression of CD25 (Supplementary Figure 1-3). We found that CD25^+^ and CD25^−^ Foxp3^+^CD4^+^ Tregs showed similar tendency as Foxp3^+^CD4^+^ Tregs during both, steady state and colitis conditions. Next we analyzed natural and inducible CD4^+^ Tregs in the spleen, colon as well as MLN after DSS treatment. To distinguish the subset of CD4^+^ Tregs, we used Helios expression suggesting natural CD4^+^ Tregs specifically express Helios but inducible ones do not. In the MLN of CCR7 KO mice, Helios^−^ inducible Tregs were decreased but Helios^+^ natural Tregs rather increased (Supplementary Figure 4). In the mucosal periphery such as colon, both type of Tregs were increased. To confirm whether impaired DC migration with CCR7 deficiency inhibited the generation of the effector CD4^+^ T cells, we investigated Th1/Th17 population in the MLN after DSS treatment. In the MLN of CCR7 KO mice, Th1 and Th17 populations were not decreased (Supplementary Figure 5). Instead, Th1 population was increased in the MLN of CCR7 KO mice. These result suggested that low inflammation to CCR7 deficiency was not from decreased generation of effector CD4^+^ T cells following impaired DC migration. Consistent with previous reports, our result showed that the lack of CCR7 impaired lymph node homing of CD4^+^ Tregs from peripheral tissue, which is otherwise essential for their suppressive function. However, we could not completely rule out the possibility that the accumulated CD4^+^ Tregs in the periphery could still exert suppressive effect on the site of intestinal inflammation.

### CCR7KO CD8^+^ T cells in the lamina propria of the gut express higher level of IL-10 than those in the WT mice

**Figure 4:**
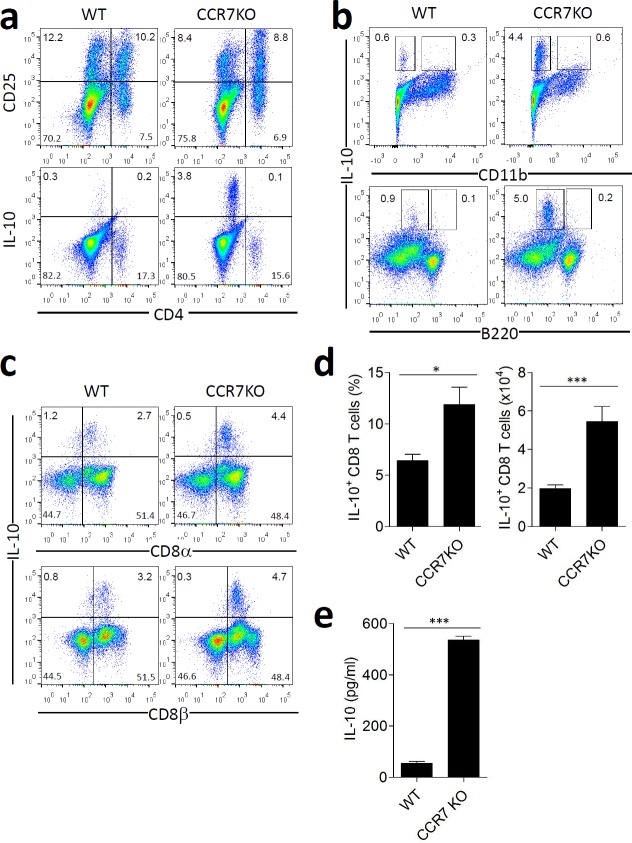
CCR7KO CD8^+^ T cell in the lamina propria of gut express higher level of IL-10 At Day 8 of DSS-induced colitis, lymphocytes and IL-10-secreting cells were analyzed from the colon. **a.** CD25 expression and IL-10 secretion by CD4^+^ T cells, **b.** IL-10 secretion on CD11b^+^ myeloid cells and B220^+^ B cells, **c.**-**d.** IL-10 secretion by lamina propria CD8^+^ T cells, Student's *t*-test, *p < 0.05, ***p < 0.001 **e.** IL-10 production by CD8^+^ T cells isolated from colon of WT or CCR7KO mice. Student's *t*-test, ***p < 0.001.

The colon in CCR7KO mice treated with DSS showed less inflammation, despite the absence of CCR7, in circumstances that increase the risk of overwhelming activation of immune responses. This finding suggests that immune suppressive factors other than CD4^+^ Tregs may be involved. IL-10 is a representative cytokine that has a regulatory role in gut immunity [18]. Therefore, we assessed IL-10 secretion from immune cells in the colon. At Day 8 of DSS-induced colitis, the percentage of CD4^+^ T cells expressing CD25^+^ (CD25^+^CD4^+^ T cells), which consisted of effector T cells and Tregs, in the colon of WT mice was similar to that of CCR7KO mice (Figure 4a). However, we did not observe significant secretion of IL-10 from CD4^+^ T cell populations comprising of Foxp3^+^CD4^+^ Tregs, whereas there was significant level of IL-10 secretion from CD4^−^ cell population obtained from the colon of CCR7KO mice. To further identify cells secreting IL-10, we analyzed other cells from the mouse LP. We found that IL-10 was not secreted by CD11b^+^ cells comprising macrophages and some myeloid DCs as well as B220^+^ cells comprising of B cells and plasmacytoid DCs (Figure 4b). Interestingly, we could find that IL-10 expressing CD8α^+^CD8β^+^ T cells were isolated from colon LP in both, WT and CCR7KO mice (Figure 4c and 4d), at a higher quantity in CCR7KO as compared to WT. IL-10 levels from culture supernatant of colon CD8^+^ T cells were measured. IL-10 secretion of CCR7KO CD8^+^ T cells were significantly enhanced compared to that of WT CD8^+^ T cells (Figure 4e). CD8^+^ intraepithelial lymphocytes isolated from colon could not produce IL-10 (Supplementary Figure 6). These results suggest that CCR7KO immune cells expressed more IL-10 and the main IL-10-producing cells were CD8^+^ T cells in the colon LP.

### CCR7KO CD8^+^ T cells showed a regulatory effect on CD4^+^ T cells

**Figure 5:**
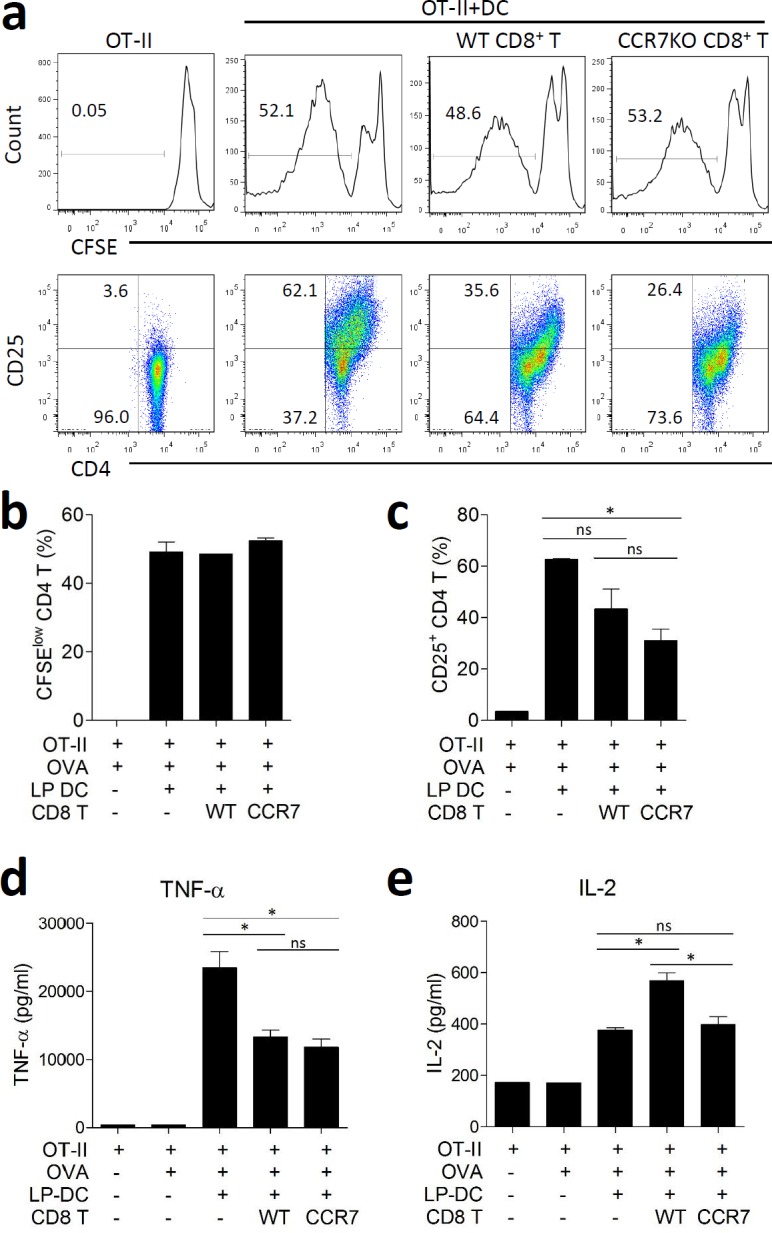
CCR7KO CD8^+^ T cells show regulatory effect on CD4^+^ T cells OT-II CD4^+^ T cells from naïve OT-II spleen and CD11c^+^ DCs isolated from the intestine were incubated with 200 μg/ml OVA in the presence of colon CD8^+^ T cells with DSS-induced colitis. **a.** CFSE dilution and CD25 expression on OT-II CD4^+^ T cells, representative data from 5 repeated experiments, **b.** the proliferation of OT-II CD4^+^ T cells was analyzed *via* CFSE dilution, **c.** the activation of OT-II CD4^+^ T cells was analyzed in terms of CD25 expression. At day 5 of culture, the culture supernatants were analyzed for cytokine production, **d.** TNF-α and **e.** IL-2. ns, not significant; *p < 0.05; one-way ANOVA.

We investigated whether IL-10-producing CD8^+^ T cells had a regulatory function. When OT-II CD4^+^ T cells were co-cultured with intestinal CD11c^+^ DCs in presence of their cognate antigen, OVA, the OT-II CD4^+^ T cells successfully proliferated and were activated, as assessed by CFSE dilution and CD25 expression, respectively (Figure 5a-5c). Isolated colon CD8^+^ T cells during DSS-induced colitis were added to the culture for activation of the OT-II CD4^+^ T cells. Both, WT and CCR7KO CD8^+^ T cells did not significantly inhibit the proliferation of the OT-II CD4^+^ T cells (Figure 5a and 5b). However, there was a significant reduction in CD25 expression by CD4^+^ T cells when they were incubated with CCR7KO CD8^+^ T cells, whereas the expression of CD25 was only slightly decreased in WT CD8^+^ T cells (Figure 5a and 5c). Further, these CD8^+^ T cells suppressed TNF-α secretion in the CD4^+^ T cell culture system (Figure 5d). However, IL-2 was rather increased during co-culture with WT CD8^+^ T cells, but not CCR7KO CD8^+^ T cells (Figure 5e). These results suggest that IL-10-producing colon CD8^+^ T cells had a regulatory effect on CD4^+^ T cells but their regulation was implemented as subtle way without inhibition of CD4^+^ T cell proliferation.

**Figure 6:**
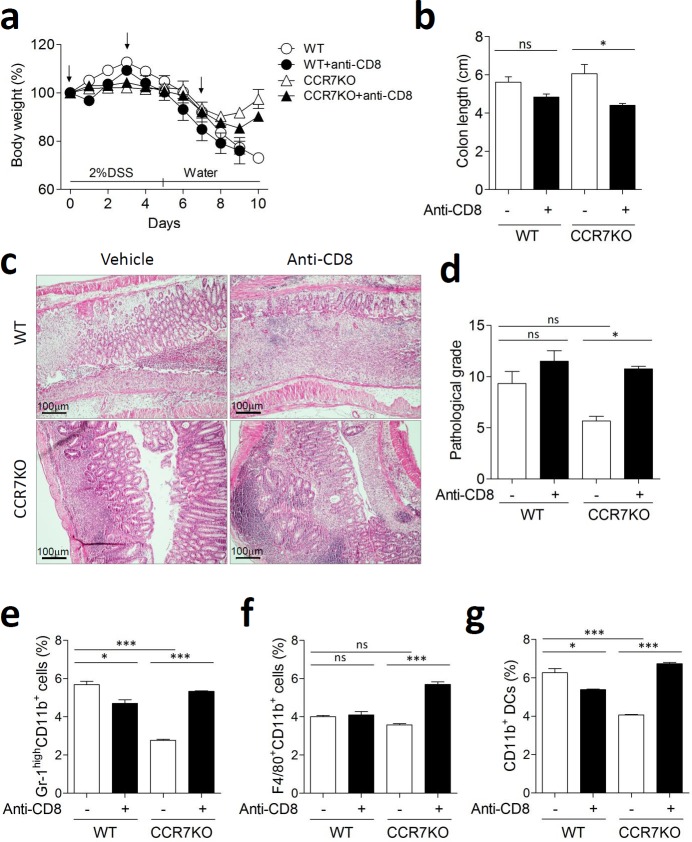
CCR7KO mice showed exacerbated colitis in absence of CD8^+^ T cells Mice were treated with 2% DSS for 5 days followed by switching to drinking water. Anti-CD8 antibody was treated tree times at indicated time point (arrow). At day 10 of DSS-induced colitis, inflammation was analyzed from the colon (*n* = 4). **a.** body weight changes, **b.** colon length; ns, not significant; *p < 0.05; one-way ANOVA, **c.** representative H&E images of distal colon (*n* = 4), **d.** pathological grade from histological examination of colon stained with H&E; Student's *t*-test, **e.** Gr-1^high^CD11b^+^ neutrophil population analysis, **f.** F4/80^+^CD11b^+^ macrophages analysis, **g.** dendritic cell analysis. Student's *t*-test.

To convince the role of IL-10 producing CD8^+^ T cells for immune regulation, we investigated colitis following depletion of CD8^+^ T cells using three times treatment of anti-CD8 antibody. Depletion of CD8^+^ T cells was confirmed by analyzing peripheral blood mononuclear cells and colon (Supplementary Figure 7). In absence of CD8^+^ T cells, CCR7KO mice showed slightly aggravated weight loss (Figure 6a). Although there was no significant difference of colon length in WT, the colon length of CCR7KO mice in absence of CD8^+^ cells was significantly decreased than that of CCR7KO mice having CD8^+^ cells (Figure 6b). Further, histological examination of colon showed that CCR7KO mice showed significantly severe inflammatory pathology in absence of CD8^+^ T cells after DSS treatment (Figure 6c and 6d). Inflammatory innate cells such as neutrophil, macrophages and myeloid dendritic cells in the colon of CCR7KO mice were obviously increased in absence of CD8^+^ T cells (Figure 6e-6g). These results suggested that intestinal inflammation was exacerbated in the CCR7KO mice when CD8^+^ T cells were depleted although the role of CD8^+^ T cells was ambiguous in the WT mice. Collectively, these results suggest that CCR7 deficiency increases IL-10 production of CD8^+^ T cells and then causes suppression of intestinal inflammation.

### Plasmacytoid DCs in the gut LP increased and had an immune regulatory function in the absence of CCR7

**Figure 7:**
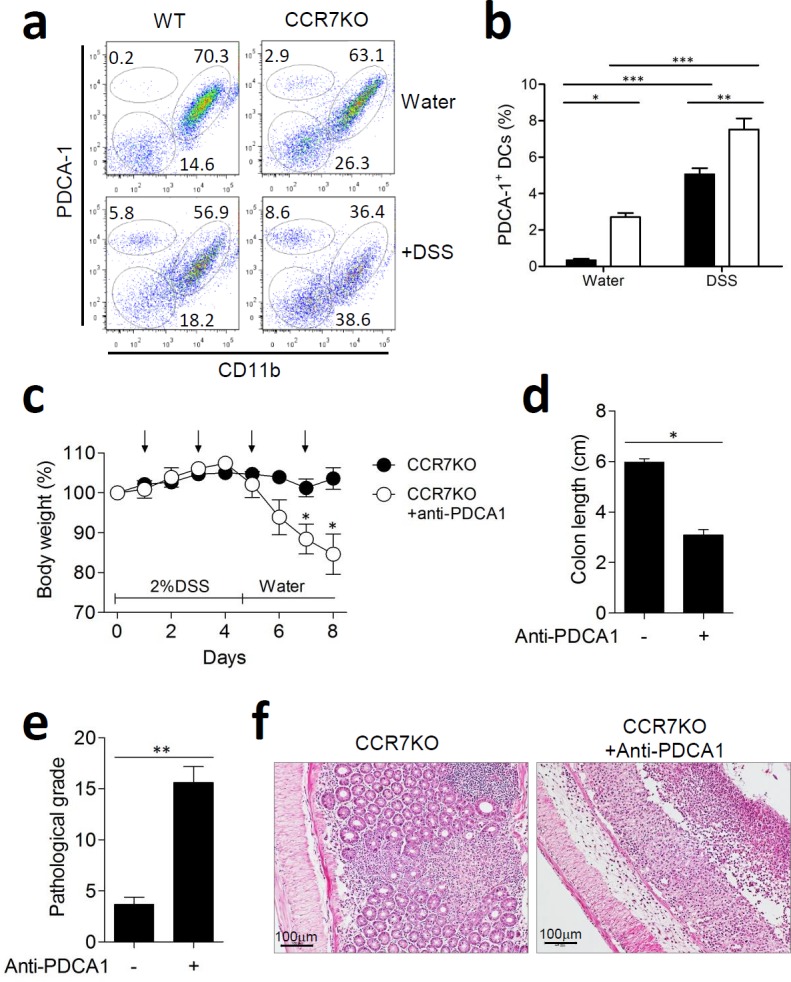
Plasmacytoid DCs in the lamina propria of gut expanded and had an immune regulatory role in the absence of CCR7 At Day 9 of DSS-induced colitis, DC subsets from the colon were analyzed. **a.** PDCA-1^+^ plasmacytoid DC *versus* CD11b^+^ DCs, representative data from 3 repeated experiments, **b.** percentage of DC subsets among CD11c^+^ DCs in the colon. *p < 0.05, **p < 0.01, ***p < 0.001 compared with matched control group using one-way ANOVA. Mice were treated with 2% DSS for 5 days and anti-PDCA1 antibody was treated every other day (arrow). At day 9 of DSS-induced colitis, inflammation was analyzed from the colon (*n* = 5). **c.** body weight changes, **d.** colon length; *p < 0.05; Student's t-test, (c) pathological grade from histological examination of colon stained with H&E; **p < 0.01; Student's *t*-test, (d) representative H&E images of distal colon (*n* = 5).

In the intestine, plasmacytoid DCs (pDCs) are known to play an immuno-suppressive role by inducing oral tolerance [19]. Subsets of CD11c^+^ DCs in the colon LP were analyzed at Day 9 of DSS-induced colitis by using plasmacytoid dendritic cell antigen-1 (PDCA-1) as a marker for pDCs in order to determine any change in the pDC subsets. Thus, we identified 3 subsets of CD11c^+^ DCs: PDCA-1^+^CD11b^−^ pDCs, PDCA-1^low^CD11b^+^ myeloid DCs, and PDCA-1^−^CD11b^−^ DCs. At steady state, the percentage of pDCs was lower in both, WT and CCR7KO groups, as compared to those after DSS treatment, although the CCR7KO group had higher numbers and proportion of pDCs than the WT group (Figure 7a-7b, Supplementary Figure 8). When treated with DSS, pDCs increased in the colon of both, WT and CCR7KO mice. Interestingly, CCR7 deficiency resulted in a higher percentage of pDCs as compared to that in the WT mice, in the DSS-induced colitis model. CD11b^+^ DCs were slightly but not significantly decreased during intestinal inflammation.

To investigate the role of increased pDCs for immune regulation, mice were administered with 2% DSS and pDCs were depleted by anti-PDCA1 antibody. We confirmed that pDCs in colon were considerably reduced following treatment of anti-PDCA1 antibody (Supplementary Figure 9). In absence of pDCs, CCR7KO mice showed significantly aggravated weight loss (Figure 7c). In addition, the colon length of CCR7KO mice was significantly decreased by the pDCs depletion (Figure 7d). Further, histological examination of colon showed that CCR7KO mice showed significantly severe inflammatory pathology in absence of pDCs after DSS treatment (Figure 7e and 7f). These results suggested that intestinal inflammation was exacerbated in the CCR7KO mice with reduced pDCs. Collectively, these results suggest that increased pDCs in large intestine of CCR7KO mice have regulatory effects against DSS-induced colitis.

## DISCUSSION

The intestinal immune system has evolved a regulatory system involving CD4^+^ Tregs expressing Foxp3 to prevent unnecessary immune reactions to harmless external stimuli such as commensals and food. In order to exert their regulatory function, Tregs need to migrate from the periphery to draining lymph nodes *via* the interaction of CCL19 or CCL21 with CCR7 receptors. CCR7 is highly expressed on various kinds of immune cells such as naïve and central memory T cells, B cells, and mature DCs as well as CD4^+^ Tregs [12]. Therefore, CCR7 deficiency generally results in the attenuation of immune responses due to the impaired migration of DCs and abnormal secondary lymphoid organ architecture. Despite impaired DC migration, effector T cells including both CD4^+^ and CD8^+^ T cells rather increased in the peripheral tissue in the CCR7KO mice which exacerbated disease condition for airway allergy and nephrotoxic serum nephritis in the CCR7KO mice [20, 21]. Therefore, impaired migration of Treg and peripheral accumulation of effector T cells could lead to hyper-immune responses in CCR7KO mice. Therefore, CCR7 deficiency was expected to cause functional defect of CD4^+^ Tregs and elicit severe intestinal pathology in response to inflammatory stimuli.

Unexpectedly, in this study we found that CCR7KO mice showed less severe inflammation in the gut as compared to WT mice in a DSS-induced colitis mouse model. Therefore, we analyzed other regulatory factors to explain the attenuated intestinal inflammation of CCR7KO mice. IL-10, an immunosuppressive cytokine is produced by CD8^+^ and not CD4^+^ T cells in the colon of CCR7KO mice. These IL-10-producing CD8^+^ T cells inhibited the activation of CD4^+^ T cells and the subsequent TNF-α secretion. In addition, regulatory pDCs were also significantly increased in CCR7KO colon during colitis. Depletion of CD8^+^ T cells and pDCs exacerbated colitis in CCR7KO mice. These results suggested that regulatory CD8^+^ T cells and pDCs could maintain gut homeostasis, which compensated for attenuated Foxp3^+^CD4^+^ Tregs without CCR7.

IL-10-expressing CD8^+^ T cell numbers in the colon of CCR7KO mice were dramatically higher than those in the colon of WT mice during inflammation, although they were also found at low levels in the steady state. This might explain why colitis in CCR7KO mice was mitigated despite the defective function of Foxp3^+^ Tregs. Suppressive CD8^+^ T cells expressing IL-10 and IFN-γ were first described in the 1970s and they have a long history in immunology [22, 23]. However, the lack of potential markers for regulatory CD8^+^ T cells delayed further investigations. Non-classical MHC class Ib (Qa-1)-restricted CD8αα^+^ T cells can repress autoimmune diseases by not only direct killing of effector T cells *via* perforin but also with the help of immunosuppressive cytokines such as TGF-β and IL-10 [24, 25]. In our previous report, we identified regulatory CD8αβ^+^ T cells expressing IL-10, IL-9, IL-13, and IFN-γ in the SI, which could be secreted by non-migratory CX_3_CR_1_^+^ phagocytic cells independent of MLN [26]. These CD8^+^ T cells inhibited the activation and differentiation of inflammatory CD4^+^ T cells *via* IL-10 expression in the colitis model. Collectively, CD8αβ^+^ T cells expressing IL-10 were enhanced when the function of CD4^+^ Tregs was reduced and thus played a compensatory role in the gut to bring about immune regulation.

IL-10 is one of the most common cytokines implicated in the maintenance of intestinal homeostasis. IL-10^−/−^ mice developed spontaneous, progressive colitis, which eventually affected the small intestine as well [27, 28]. IL-10 acts not only to control effector T cell function, but also to suppress the proinflammatory activity of myeloid cells, since the IL-10 receptor is expressed on both innate and adaptive immune cells as well as on non-hematopoietic cells [29]. STAT3 protein is essential for both, the anti-proliferative and the anti-inflammatory effects of IL-10 on macrophages [30]. IL-10 also reduces the expression of co-stimulatory surface molecules on macrophages [31]. Although IL-10 achieves control of adaptive immune response indirectly *via* antigen-presenting cell signaling, IL-10 directly promotes the maintenance, expansion, and function of Treg cells as well, *via* the IL-10 receptor [32]. Diverse cellular sources of IL-10 have been identified in the gut, including several different subsets of CD4^+^ T cells, including Foxp3^+^ and Foxp3^−^ cells [33, 34] as well as CD8^+^ T cells. CD8^+^ T cells cultured in the presence of IL-4 acquire an IL-10-producing phenotype and after adoptive transfer, these cells are capable of amelioration of colitis [35]. In addition, IL-10-producing CD8αβ^+^ T cells in the LP and epithelium of SI were produced by CX_3_CR_1_^hi^ LP macrophages [26]. Additionally, B cells are a functional source of IL-10 [36], as are CX_3_CR_1_^hi^ LP macrophages [37-39]. In this study, the main cellular source of IL-10 in the inflamed CCR7KO colon was revealed as CD8αβ^+^ T cells, which appear to be a major player in immune regulation in the absence of intact Tregs.

pDCs, unlike conventional DCs, express B220 and PDCA-1 [40] in addition to low levels of CD11c, CD8α^+^, and CD11b^−^. pDCs are known to play a crucial role in innate immunity against viral infection by producing type-I interferons including IFN-α and IFN-β [41]. In the gut, they interestingly have a regulatory function during conditions such as oral tolerance [42]. pDCs mediate anti-inflammatory responses subsequent to TLR2-microbial polysaccharide A engagement [43]. Type-I interferon secreted by pDCs has shown to support CD4^+^ Treg function and repress colitis [44, 45]. During DSS-induced colitis, significantly higher numbers of pDCs were present in the colon of CCR7KO mice than that in WT mice. Exacerbated colitis by the depletion of pDCs suggested that these pDCs exist in colon were involved in the maintenance of intestinal immune homeostasis. However, it is unclear if these pDCs of CCR7KO mice had any direct or indirect influence on IL-10-producing CD8^+^ T cells.

There is no doubt that Foxp3^+^CD4^+^ Tregs are master regulators of immune homeostasis. The intestinal environment is one of the most significant sites for immune suppression in our body. In fact, the proportion of Foxp3^+^CD4^+^ Tregs amongst peripherally circulating CD4^+^ T cells is highest in the gut, especially the colon. However, functional defect of Foxp3^+^CD4^+^ Tregs does not lead to drastic pathological disease in response to inflammatory stimuli since there are alternatives, such as CD8^+^ Tregs and regulatory DCs who take over when the need arises. Collectively, these results suggest that the intestine contains an intricate regulatory mechanism to maintain immune homeostasis.

## MATERIALS AND METHODS

### Mice

All experiments were approved by the Institutional Animal Care and Use Committee of Kangwon National University and Ajou University. WT C57BL/6 mice were purchased from Charles River Laboratories (Orient Bio Inc., Sungnam, Korea). CCR7^−/−^ mice (C57BL/6 background) were purchased from Jackson Laboratories (Bar Harbor, ME). All mice used in the study were at 6 weeks of age. The mice were kept in the Animal Center for Pharmaceutical Research at Kangwon National University and Ajou University.

### Colitis induction

To induce colitis in mice, 2% DSS (MP Biomedicals, Solon, OH) in autoclaved drinking water were treated for 5days and then changed into drinking water without DSS. For depletion of CD8^+^ T cells, hybridomas producing depleting anti-CD8 (2.43) mAb were obtained from the ATCC and mice were treated three times as previously described [46]. For depletion of pDCs, anti-PDCA-1 antibody (eBiosciences) was treated every other day from day 1.

### Cell isolation

To isolate mononuclear cells, the spleen (SP), peripheral lymph nodes (PLN), mesenteric lymph nodes (MLN), and Peyer's Patches (PPs) were homogenized and treated with RBC lysis buffer (Sigma-Aldrich, St. Louis, MO), as necessary. To obtain mononuclear cells from the lamina propria (LP) of the small intestine (SI) and colon, the intestine was inverted onto polyethylene tubes (Becton Dickinson, Franklin Lakes, NJ) and washed 3 times with calcium- and magnesium-free phosphate-buffered saline (PBS). The intestines were treated with 1 mM dithiothreitol (DTT; Sigma-Aldrich, St. Louis, MO) to remove mucus, 30 mM EDTA to remove epithelial cells, and finally digested with 108 U/ml (for colon) or 36 U/ml (for SI) type IV collagenase (Sigma-Aldrich) for 90 min at 37°C. LP cells were then treated with discontinuous density gradient containing 66% and 44% Percoll (GE Healthcare Life sciences, Uppsala, Sweden) for T cells and Opti-prep (Axis-Shield, Oslo, Norway) with fetal bovine serum (FBS) layered on top for DCs. To evaluate IL-10 production from colon CD8^+^ T cells, CD8^+^ T cells were purified using CD8α^+^ MicroBeads (Miltenyi Biotec, Auburn, CA) and then stimulated with anti-CD3/anti-CD28 antibody for overnight. The IL-10 level of culture supernatant were measured by ELISA (eBioscience, San Diego, CA).

### Histology

The colons were washed with PBS and fixed in 4% formaldehyde for 1 h at 4°C. The tissues were dehydrated by gradually soaking them in alcohol and xylene and then embedded in paraffin. The paraffin-embedded specimens were cut into 5-μm sections, stained with hematoxylin and eosin, and viewed with a digital light microscope DS-Fi2 (Nikon, Tokyo, Japan).

### Flow cytometry

Isolated cells were incubated in 2% FBS and Fc block (BD Bioscience) for 20 min at 4°C and then stained with fluorescent-conjugated antibody. Most Abs used for flow cytometry analysis were purchased from BD Biosciences except for anti-mouse F4/80-PE (eBioscience), anti-mouse/rat Foxp3-APC (eBioscience), and anti-mouse CD317(BST2, PDCA-1)-PE (eBioscience). Data were obtained using FACSCanto (BD Bioscience) and analyzed with FlowJo software (Tree Star, Ashland, OR). For intracellular staining for IL-10, the cells were stimulated with PMA/ionomycin or anti-CD3 antibody and then fixing and permeabilization buffer were used as per manufacturer instructions.

### T cell inhibition assay

OT-II CD4^+^ T cells were isolated from the spleen of OT-II mice by using the CD4^+^ T cell Isolation Kit II (Miltenyi Biotec). OT-II CD4^+^ T cells were labeled with 1 μM CFSE (Molecular probe, Eugene, OR). CD8^+^ T cells were isolated from the colon and purified using CD8α^+^ MicroBeads (Miltenyi Biotec) as per the manufacturer instructions. CD11c^+^ DCs were isolated from the SI and further prepared using CD11c^+^ MicroBeads (Miltenyi Biotec) as per manufacturer instructions. The purity following bead isolation was >90%. Subsequently, 2 × 10^5^ OT-II CD4^+^ T cells and 1×10^5^ DCs were co-cultured with 200 μg/ml OVA in presence of 2 × 10^5^ WT or CCR7KO CD8^+^ T cells for 5 days. The CFSE level and CD25 expression on OT-II CD4^+^ T cells were analyzed by flow cytometry. The culture supernatants were analyzed for cytokine production using murine inflammatory CBA kit (BD biosciences) as per manufacturer instructions.

### Statistics

Student's *t*-test was used to compare differences between the two groups. To compare multiple groups, we performed one-way ANOVA followed by Tukey's post hoc test. For survival rate, Log-rank (Mantel-Cox) test were used. Values of *p* < 0.05 were considered to be significant.

## SUPPLEMENTARY MATERIAL FIGURES



## References

[1] Hooper LV, Littman DR, Macpherson AJ (2012). Interactions between the microbiota and the immune system. Science.

[2] Vaishnava S, Yamamoto M, Severson KM, Ruhn KA, Yu X, Koren O, Ley R, Wakeland EK, Hooper LV (2011). The antibacterial lectin RegIIIgamma promotes the spatial segregation of microbiota and host in the intestine. Science.

[3] Palm NW, de Zoete MR, Cullen TW, Barry NA, Stefanowski J, Hao L, Degnan PH, Hu J, Peter I, Zhang W, Ruggiero E, Cho JH, Goodman AL, Flavell RA (2014). Immunoglobulin A coating identifies colitogenic bacteria in inflammatory bowel disease. Cell.

[4] Fukuda S, Toh H, Hase K, Oshima K, Nakanishi Y, Yoshimura K, Tobe T, Clarke JM, Topping DL, Suzuki T, Taylor TD, Itoh K, Kikuchi J, Morita H, Hattori M, Ohno H (2011). Bifidobacteria can protect from enteropathogenic infection through production of acetate. Nature.

[5] Maloy KJ, Powrie F (2011). Intestinal homeostasis and its breakdown in inflammatory bowel disease. Nature.

[6] Kaser A, Zeissig S, Blumberg RS (2010). Inflammatory bowel disease. Annu Rev Immunol.

[7] Weaver CT, Elson CO, Fouser LA, Kolls JK (2013). The Th17 pathway and inflammatory diseases of the intestines, lungs, and skin. Annu Rev Pathol.

[8] Josefowicz SZ, Lu LF, Rudensky AY (2012). Regulatory T cells: mechanisms of differentiation and function. Annu Rev Immunol.

[9] Curotto de Lafaille MA, Lafaille JJ (2009). Natural and adaptive foxp3+ regulatory T cells: more of the same or a division of labor?. Immunity.

[10] Uhlig HH, Coombes J, Mottet C, Izcue A, Thompson C, Fanger A, Tannapfel A, Fontenot JD, Ramsdell F, Powrie F (2006). Characterization of Foxp3+CD4+CD25+ and IL-10-secreting CD4+CD25+ T cells during cure of colitis. J Immunol.

[11] Boehm F, Martin M, Kesselring R, Schiechl G, Geissler EK, Schlitt HJ, Fichtner-Feigl S (2012). Deletion of Foxp3+ regulatory T cells in genetically targeted mice supports development of intestinal inflammation. BMC Gastroenterol.

[12] Forster R, Schubel A, Breitfeld D, Kremmer E, Renner-Muller I, Wolf E, Lipp M (1999). CCR7 coordinates the primary immune response by establishing functional microenvironments in secondary lymphoid organs. Cell.

[13] von Andrian UH, Mempel TR (2003). Homing and cellular traffic in lymph nodes. Nat Rev Immunol.

[14] Debes GF, Arnold CN, Young AJ, Krautwald S, Lipp M, Hay JB, Butcher EC (2005). Chemokine receptor CCR7 required for T lymphocyte exit from peripheral tissues. Nat Immunol.

[15] Tomura M, Honda T, Tanizaki H, Otsuka A, Egawa G, Tokura Y, Waldmann H, Hori S, Cyster JG, Watanabe T, Miyachi Y, Kanagawa O, Kabashima K (2010). Activated regulatory T cells are the major T cell type emigrating from the skin during a cutaneous immune response in mice. J Clin Invest.

[16] Schneider MA, Meingassner JG, Lipp M, Moore HD, Rot A (2007). CCR7 is required for the *in vivo* function of CD4+ CD25+ regulatory T cells. J Exp Med.

[17] Schumann M, Winter S, Wichner K, May C, Kuhl AA, Batra A, Siegmund B, Zeitz M, Schulzke JD, Lipp M, Hopken UE (2012). CCR7 deficiency causes diarrhea associated with altered ion transport in colonocytes in the absence of overt colitis. Mucosal Immunol.

[18] Kole A, Maloy KJ (2014). Control of intestinal inflammation by interleukin-10. Curr Top Microbiol Immunol.

[19] Goubier A, Dubois B, Gheit H, Joubert G, Villard-Truc F, Asselin-Paturel C, Trinchieri G, Kaiserlian D (2008). Plasmacytoid dendritic cells mediate oral tolerance. Immunity.

[20] Eller K, Weber T, Pruenster M, Wolf AM, Mayer G, Rosenkranz AR, Rot A (2010). CCR7 deficiency exacerbates injury in acute nephritis due to aberrant localization of regulatory T cells. J Am Soc Nephrol.

[21] Kawakami M, Narumoto O, Matsuo Y, Horiguchi K, Horiguchi S, Yamashita N, Sakaguchi M, Lipp M, Nagase T (2012). The role of CCR7 in allergic airway inflammation induced by house dust mite exposure. Cell Immunol.

[22] Cantor H, Shen FW, Boyse EA (1976). Separation of helper T cells from suppressor T cells expressing different Ly components. II. Activation by antigen: after immunization, antigen-specific suppressor and helper activities are mediated by distinct T-cell subclasses. J Exp Med.

[23] Tsoi MS, Storb R, Dobbs S, Kopecky KJ, Santos E, Weiden PL, Thomas ED (1979). Nonspecific suppressor cells in patients with chronic graft-vs-host disease after marrow grafting. J Immunol.

[24] Hu D, Ikizawa K, Lu L, Sanchirico ME, Shinohara ML, Cantor H (2004). Analysis of regulatory CD8 T cells in Qa-1-deficient mice. Nat Immunol.

[25] Sarantopoulos S, Lu L, Cantor H (2004). Qa-1 restriction of CD8+ suppressor T cells. J Clin Invest.

[26] Chang SY, Song JH, Guleng B, Cotoner CA, Arihiro S, Zhao Y, Chiang HS, O'Keeffe M, Liao G, Karp CL, Kweon MN, Sharpe AH, Bhan A, Terhorst C, Reinecker HC (2013). Circulatory antigen processing by mucosal dendritic cells controls CD8(+) T cell activation. Immunity.

[27] Berg DJ, Davidson N, Kuhn R, Muller W, Menon S, Holland G, Thompson-Snipes L, Leach MW, Rennick D (1996). Enterocolitis and colon cancer in interleukin-10-deficient mice are associated with aberrant cytokine production and CD4(+) TH1-like responses. J Clin Invest.

[28] Kuhn R, Lohler J, Rennick D, Rajewsky K, Muller W (1993). Interleukin-10-deficient mice develop chronic enterocolitis. Cell.

[29] Moore KW, de Waal Malefyt R, Coffman RL, O'Garra A (2001). Interleukin-10 and the interleukin-10 receptor. Annu Rev Immunol.

[30] Takeda K, Clausen BE, Kaisho T, Tsujimura T, Terada N, Forster I, Akira S (1999). Enhanced Th1 activity and development of chronic enterocolitis in mice devoid of Stat3 in macrophages and neutrophils. Immunity.

[31] Ding L, Linsley PS, Huang LY, Germain RN, Shevach EM (1993). IL-10 inhibits macrophage costimulatory activity by selectively inhibiting the up-regulation of B7 expression. J Immunol.

[32] Hadis U, Wahl B, Schulz O, Hardtke-Wolenski M, Schippers A, Wagner N, Muller W, Sparwasser T, Forster R, Pabst O (2011). Intestinal tolerance requires gut homing and expansion of FoxP3+ regulatory T cells in the lamina propria. Immunity.

[33] Kamanaka M, Huber S, Zenewicz LA, Gagliani N, Rathinam C, O'Connor W, Wan YY, Nakae S, Iwakura Y, Hao L, Flavell RA (2011). Memory/effector (CD45RB(lo)) CD4 T cells are controlled directly by IL-10 and cause IL-22-dependent intestinal pathology. J Exp Med.

[34] Maynard CL, Harrington LE, Janowski KM, Oliver JR, Zindl CL, Rudensky AY, Weaver CT (2007). Regulatory T cells expressing interleukin 10 develop from Foxp3+ and Foxp3− precursor cells in the absence of interleukin 10. Nat Immunol.

[35] Zhao Y, Zhao H, Sun Y, Hao J, Qi X, Zhou X, Wu Z, Wang P, Kaech SM, Weaver CT, Flavell RA, Zhao L, Yao Z, Yin Z (2013). IL-4 induces a suppressive IL-10-producing CD8+ T cell population *via* a Cdkn2a-dependent mechanism. J Leukoc Biol.

[36] Mizoguchi A, Mizoguchi E, Takedatsu H, Blumberg RS, Bhan AK (2002). Chronic intestinal inflammatory condition generates IL-10-producing regulatory B cell subset characterized by CD1d upregulation. Immunity.

[37] Rivollier A, He J, Kole A, Valatas V, Kelsall BL (2012). Inflammation switches the differentiation program of Ly6Chi monocytes from antiinflammatory macrophages to inflammatory dendritic cells in the colon. J Exp Med.

[38] Chirdo FG, Millington OR, Beacock-Sharp H, Mowat AM (2005). Immunomodulatory dendritic cells in intestinal lamina propria. Eur J Immunol.

[39] Denning TL, Wang YC, Patel SR, Williams IR, Pulendran B (2007). Lamina propria macrophages and dendritic cells differentially induce regulatory and interleukin 17-producing T cell responses. Nat Immunol.

[40] Colonna M, Trinchieri G, Liu YJ (2004). Plasmacytoid dendritic cells in immunity. Nat Immunol.

[41] Martin P, Del Hoyo GM, Anjuere F, Arias CF, Vargas HH, Fernandez LA, Parrillas V, Ardavin C (2002). Characterization of a new subpopulation of mouse CD8alpha+ B220+ dendritic cells endowed with type 1 interferon production capacity and tolerogenic potential. Blood.

[42] Bilsborough J, George TC, Norment A, Viney JL (2003). Mucosal CD8alpha+ DC, with a plasmacytoid phenotype, induce differentiation and support function of T cells with regulatory properties. Immunology.

[43] Dasgupta S, Erturk-Hasdemir D, Ochoa-Reparaz J, Reinecker HC, Kasper DL (2014). Plasmacytoid dendritic cells mediate anti-inflammatory responses to a gut commensal molecule *via* both innate and adaptive mechanisms. Cell Host Microbe.

[44] Lee SE, Li X, Kim JC, Lee J, Gonzalez-Navajas JM, Hong SH, Park IK, Rhee JH, Raz E (2012). Type I interferons maintain Foxp3 expression and T-regulatory cell functions under inflammatory conditions in mice. Gastroenterology.

[45] Kole A, He J, Rivollier A, Silveira DD, Kitamura K, Maloy KJ, Kelsall BL (2013). Type I IFNs regulate effector and regulatory T cell accumulation and anti-inflammatory cytokine production during T cell-mediated colitis. J Immunol.

[46] Ko HJ, Kim YJ, Kim YS, Chang WS, Ko SY, Chang SY, Sakaguchi S, Kang CY (2007). A combination of chemoimmunotherapies can efficiently break self-tolerance and induce antitumor immunity in a tolerogenic murine tumor model. Cancer Res.

