# Supplementary material for: Compensatory roles of CD8+ T cells and plasmacytoid dendritic cells in gut immune regulation for reduced function of CD4+ Tregs
Source: Oncotarget. 2016 Feb 19;7(10):10947–61. doi: 10.18632/oncotarget.7510 (PMC4905450; doi:10.18632/oncotarget.7510)
Supplement: Supplementary file 1 [file oncotarget-07-10947-s001.pdf]

Compensatory roles of CD8^{+} T cells and plasmacytoid dendritic cells in gut immune regulation for reduced function of CD4^{+} Tregs

Supplementary Material

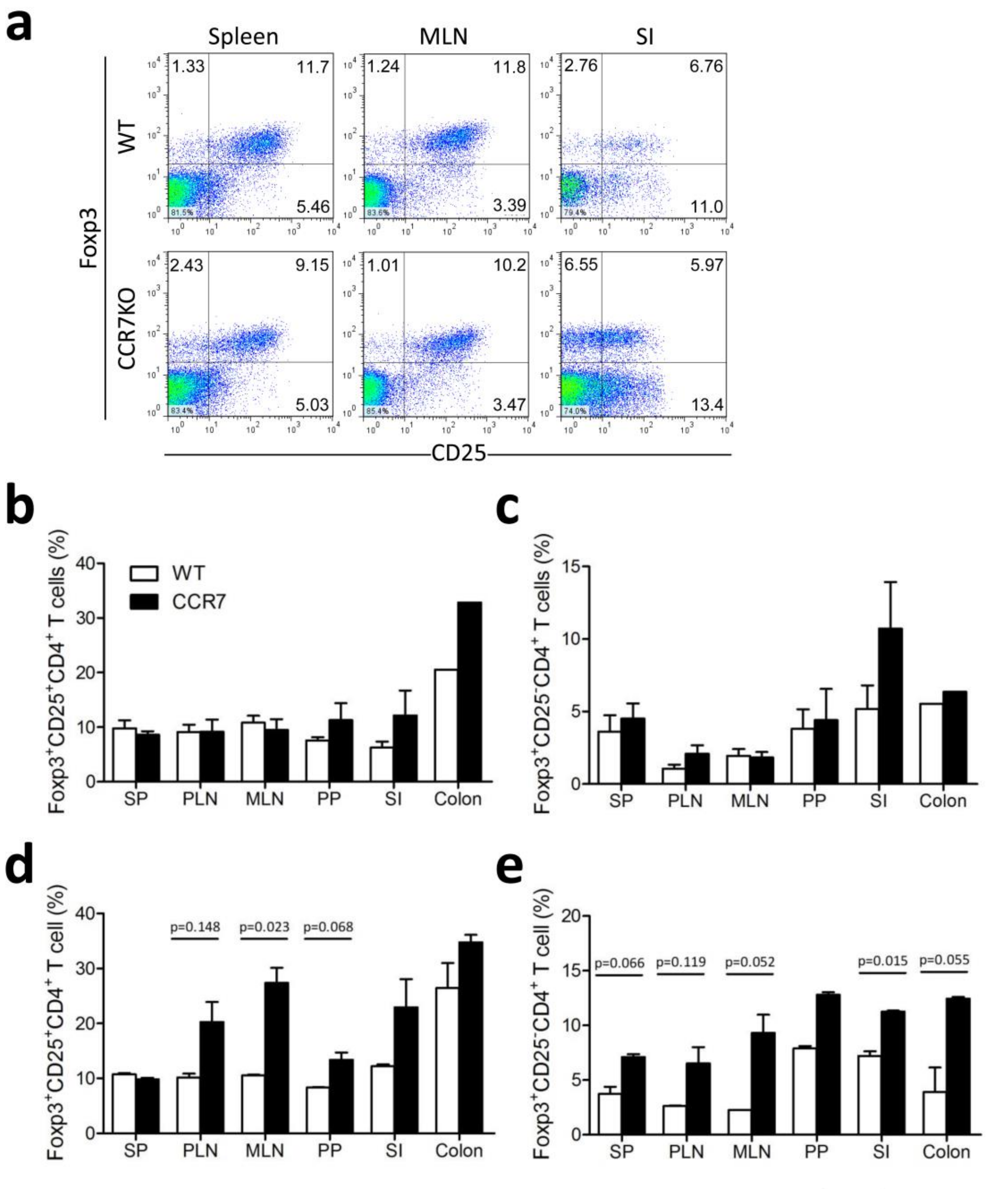

Supplementary Figure 1. The percentage of CD25 expression on Foxp3^{+}CD4^{+} Tregs was analyzed under steady-state conditions or intestinal inflammation. Foxp3^{+}CD4^{+} Tregs were analyzed from various immune tissues. (a) representative data from 5 repeated experiments, (b-c) the percentage of CD25^{+} (b) or CD25^{-}Foxp3^{+}CD4^{+}Tregs (c) among CD4^{+} T cells at steady state condition, (d-e) the percent of CD25^{+} (d) or CD25^{-}Foxp3^{+}CD4^{+}Tregs and (e) among CD4^{+} T cells at Day 8 of DSS-induced colitis.

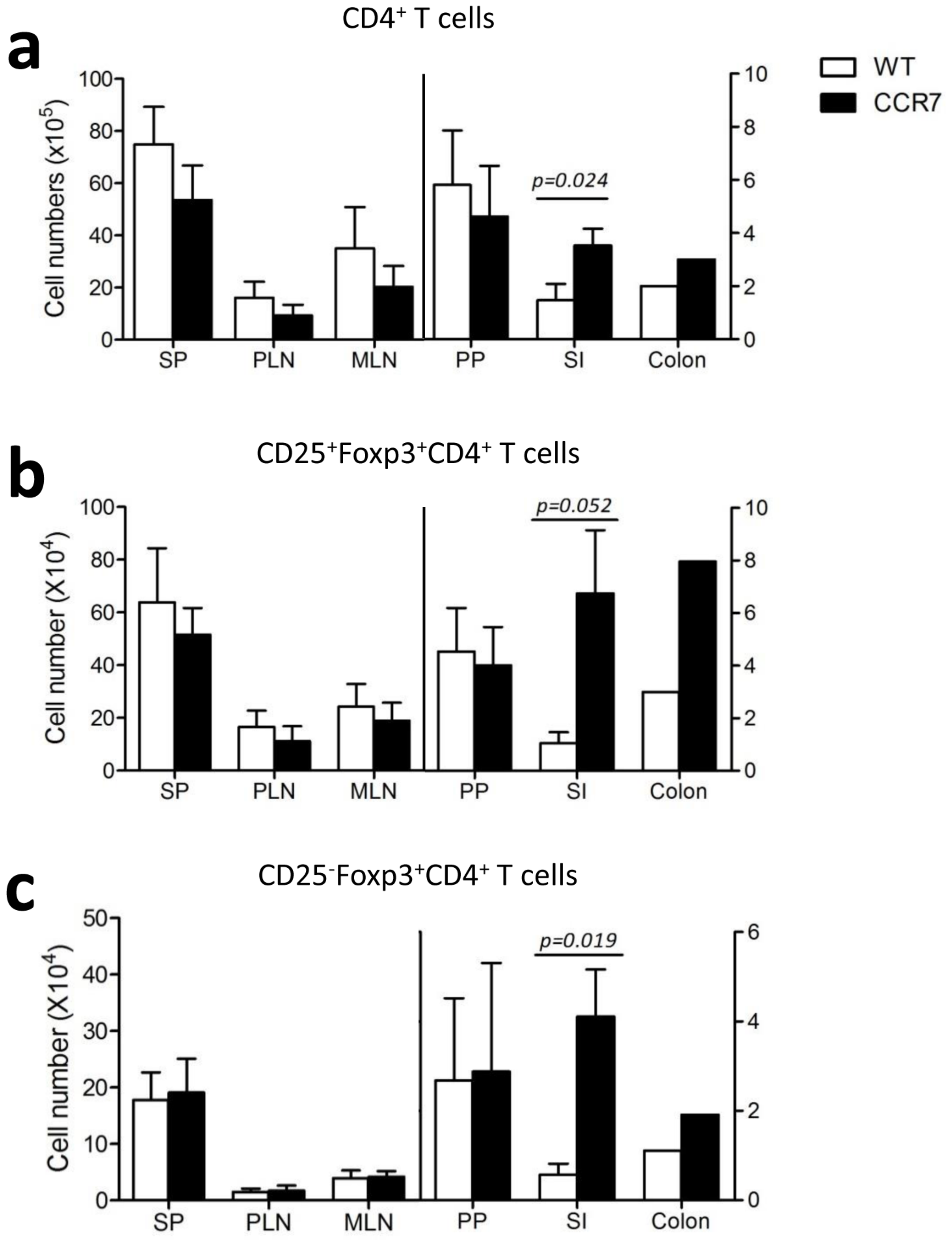

Supplementary Figure 2. Foxp3^{+}CD4^{+} Tregs accumulated in the intestine under steady-state conditions. (a) number of CD4^{+} T cells, (b) number of CD25^{+}Foxp3^{+}CD4^{+} T cells, and (c) number of CD25^{-}Foxp3^{+}CD4^{+} T cells.

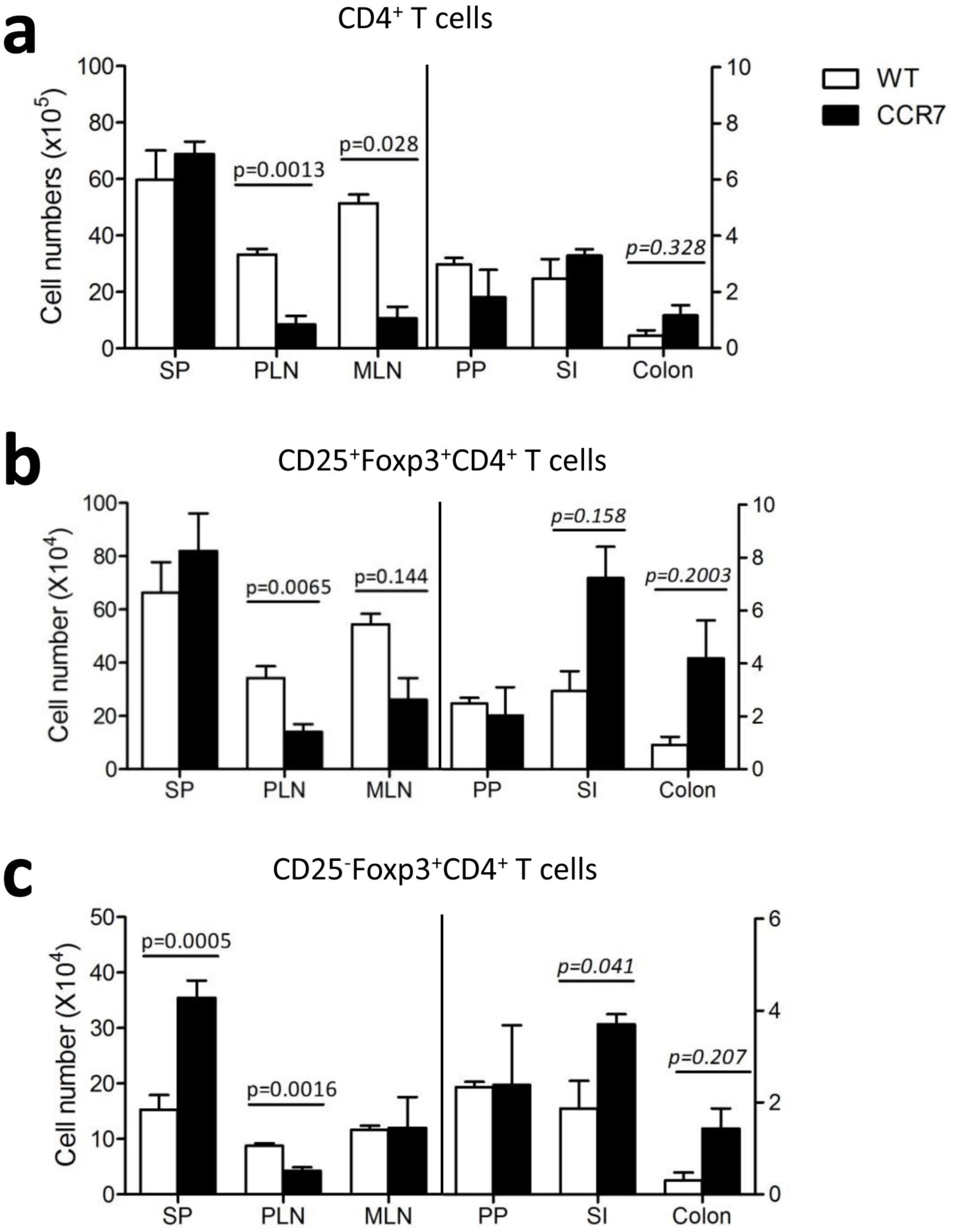

Supplementary Figure 3. Foxp3⁺CD4⁺ Tregs accumulated in the intestine under inflammatory conditions. At Day 8 of DSS-induced colitis, Foxp3⁺CD4⁺ Tregs from WT and CCR7KO mice were analyzed. (a) number of CD4⁺ T cells, (b) number of CD25⁺Foxp3⁺CD4⁺ T cells, and (c) number of CD25⁻Foxp3⁺CD4⁺ T cells.

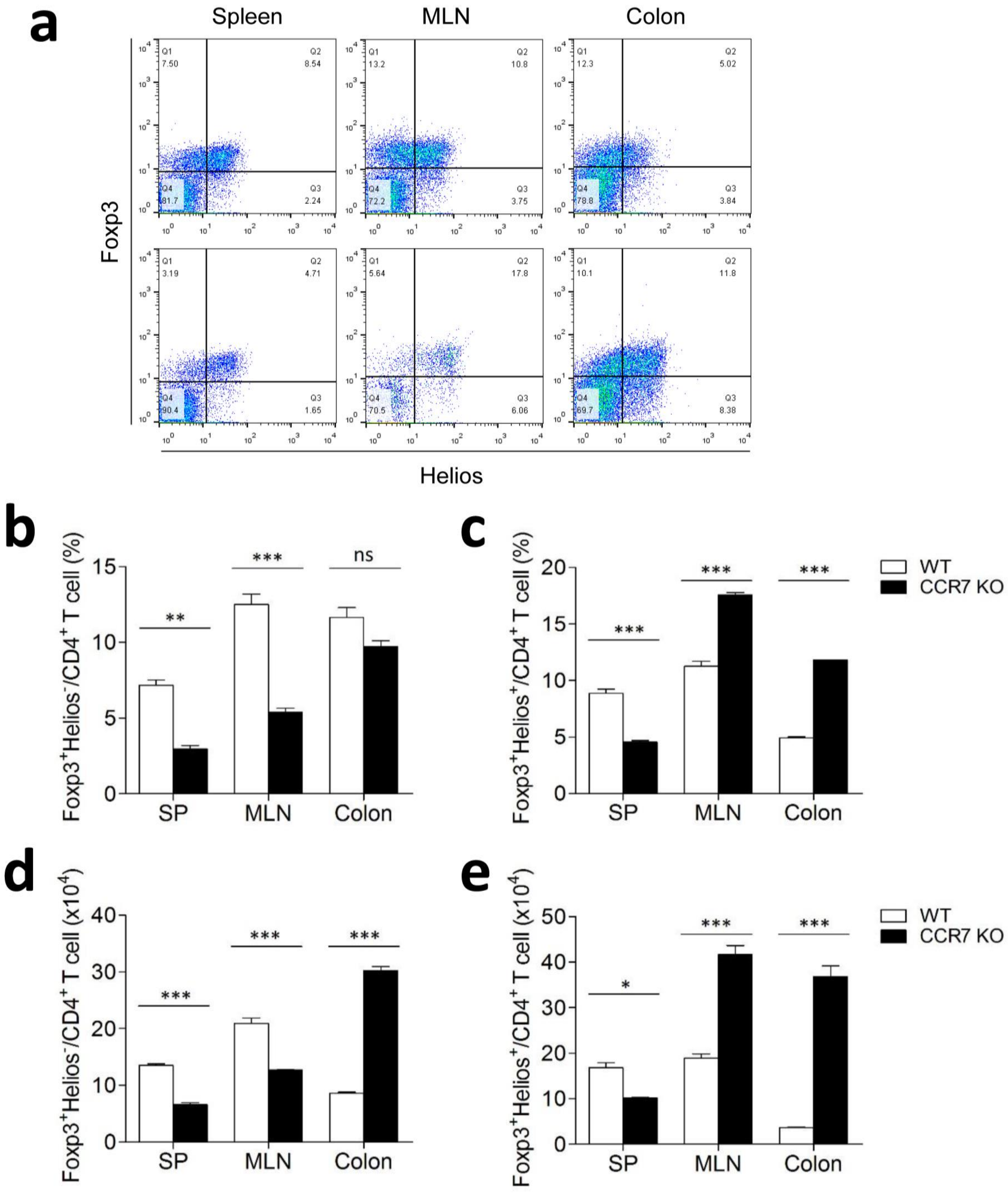

Supplementary Figure 4. Natural (Helios^{+}) and inducible (Helios^{-}) Foxp3^{+}CD4^{+} Tregs accumulated in the colon under inflammatory conditions. Foxp3^{+}CD4^{+} Tregs were analyzed from various immune tissues. (a) representative data from three repeated experiments, (b-c) the percentage of Helios^{-} (b) or Helios^{+}Foxp3^{+}CD4^{+} Tregs (c) among CD4^{+} T cells, (d-e) the absolute numbers of Helios^{-} (d) or Helios^{+}Foxp3^{+}CD4^{+} Tregs and (e) among CD4^{+} T cells at Day 8 of DSS-induced colitis.

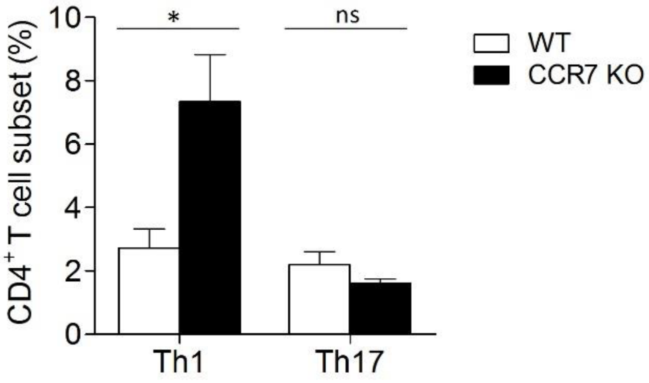

Supplementary Figure 5. The generation of helper CD4$^+$ T cells was not impaired in the gut draining lymph node of CCR7KO mice. Helper CD4$^+$ T cells including IFN-γ and IL-17A producing Th1 and Th17 were analyzed from MLN at Day 8 of DSS-induced colitis, respectively. The percentage of IFN-γ producing Th1 and IL-17A producing Th17 among CD4$^+$ T cells were summarized.

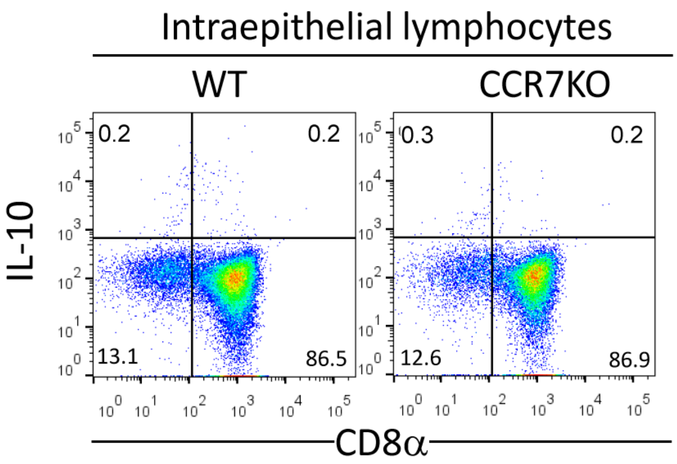

Supplementary Figure 6. IL-10 secretion by intraepithelial CD8+ T cells.

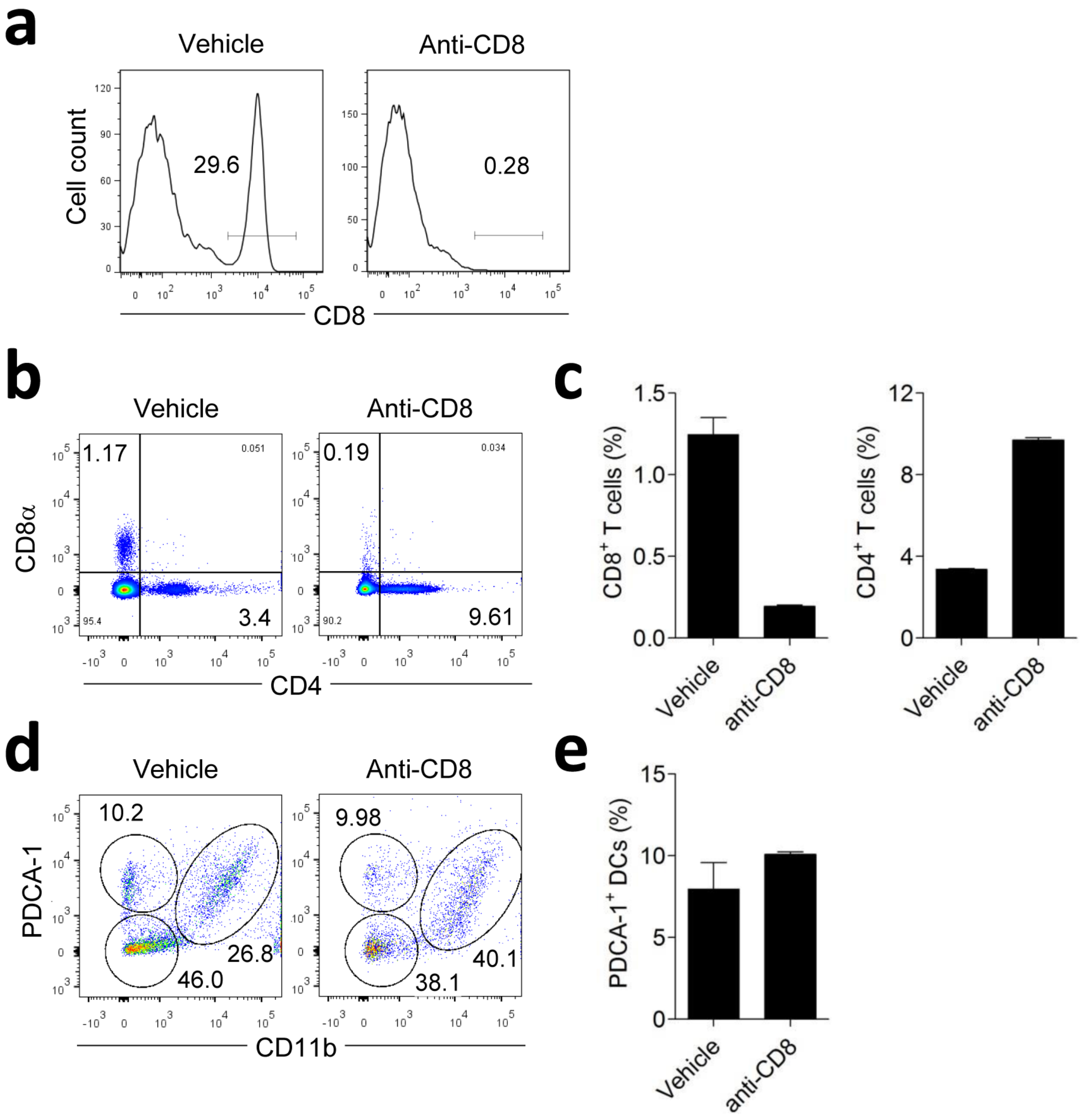

Supplementary Figure 7. Depletion of CD8$^+$ T cells using anti-CD8 antibody.

(a) Peripheral blood mononuclear cells were analyzed following treatment of anti-CD8 antibody. CD8$^+$ T cells were about 30% of CD3$^+$ population but treatment of anti-CD8 antibody could completely remove it. (b-c) CD8$^+$ T cells and (d-e) pDCs in the colon of CCR7KO mice with DSS-induced colitis were analyzed following treatment of anti-CD8 antibody.

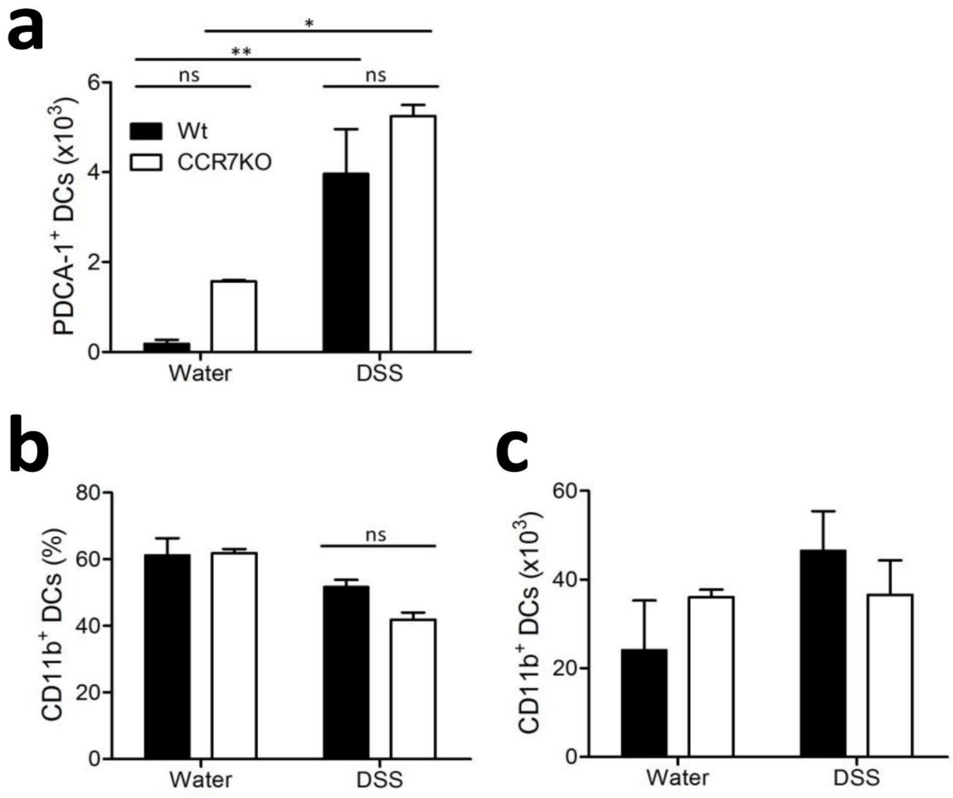

Supplementary Figure 8. Increased pDCs in the colon with DSS-induced colitis. (a) The number of PDCA-1^+ pDCs in the colon of WT and CCR7KO mice with DSS-induced colitis were analyzed. (b-c) The percentage and number of CD11b$^+$ DCs in the colon of CCR7KO mice with DSS-induced colitis were analyzed.

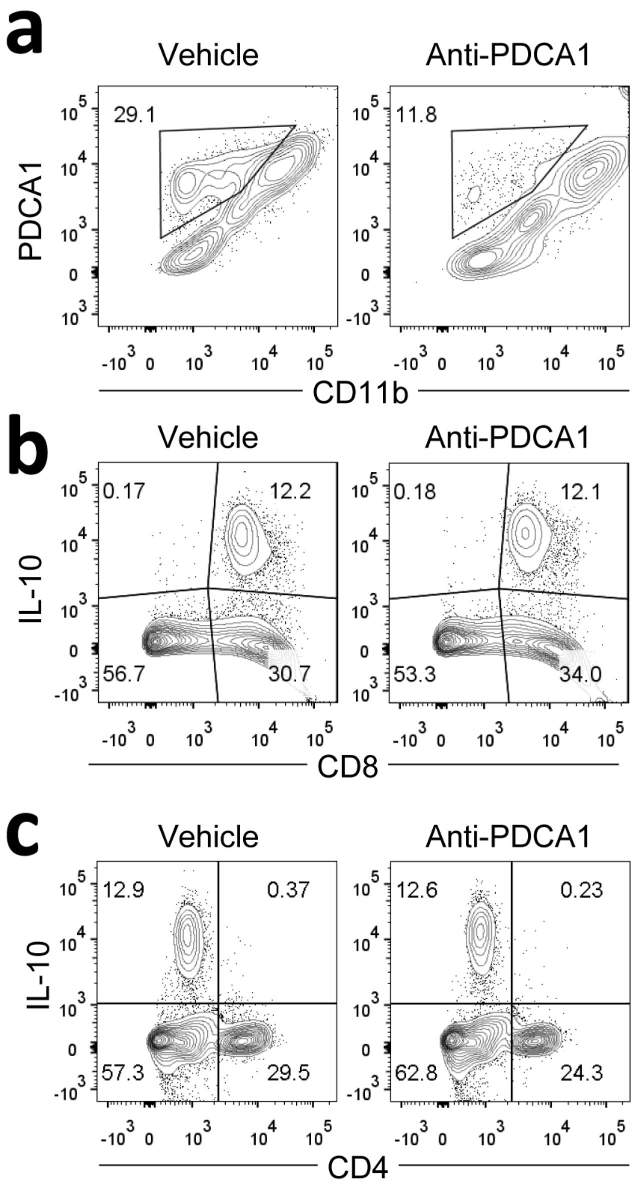

Supplementary Figure 9. Depletion of pDCs using anti-PDCA1 antibody. (a) pDCs in the colon of CCR7KO mice with DSS-induced colitis were analyzed following treatment of anti-PDCA1 antibody. (b-c) IL-10 production of $CD8^+$ and $CD4^+$ T cells in the colon of CCR7KO mice with DSS-induced colitis were analyzed following treatment of anti-PDCA1 antibody.
